# A reference genome for Colusa grass, *Neostapfia colusana*, a threatened and endangered California vernal pool plant

**DOI:** 10.1093/jhered/esaf075

**Published:** 2025-10-07

**Authors:** Lillie K Pennington, Merly Escalona, Daniel J Toews, Noravit Chumchim, Robert Cooper, Colin W Fairbairn, Mohan P A Marimuthu, Courtney Miller, Oanh H Nguyen, Dannise Ruiz-Ramos, William E Seligmann, Molly Stephens, Erin Toffelmier, H Bradley Shaffer, Rachel S Meyer, Jason P Sexton

**Affiliations:** Department of Life and Environmental Sciences, University of California, Merced, CA, United States; Genetics Department, University of Georgia, Athens, GA, United States; Department of Biomolecular Engineering, University of California Santa Cruz, Santa Cruz, CA, United States; Department of Life and Environmental Sciences, University of California, Merced, CA, United States; The Nature Conservancy, Sacramento, CA, United States; DNA Technologies and Expression Analysis Core Laboratory, Genome Center, University of California, Davis, CA, United States; Department of Ecology and Evolutionary Biology, University of California, Los Angeles, CA, United States; Department of Ecology and Evolutionary Biology, University of California, Santa Cruz, CA 95064, United States; DNA Technologies and Expression Analysis Core Laboratory, Genome Center, University of California, Davis, CA, United States; Department of Ecology and Evolutionary Biology, University of California, Los Angeles, CA, United States; DNA Technologies and Expression Analysis Core Laboratory, Genome Center, University of California, Davis, CA, United States; Department of Natural Sciences, University of Maryland Eastern Shore, Princess Anne, MD, 21853, United States; Department of Ecology and Evolutionary Biology, University of California, Santa Cruz, CA 95064, United States; Sierra Nevada Research Institute, University of California, Merced, CA, United States; Department of Ecology and Evolutionary Biology, University of California, Los Angeles, CA, United States; La Kretz Center for California Conservation Science, Institute of the Environment and Sustainability, University of California, Los Angeles, CA, United States; Department of Ecology and Evolutionary Biology, University of California, Los Angeles, CA, United States; La Kretz Center for California Conservation Science, Institute of the Environment and Sustainability, University of California, Los Angeles, CA, United States; Department of Ecology and Evolutionary Biology, University of California, Santa Cruz, CA 95064, United States; Department of Life and Environmental Sciences, University of California, Merced, CA, United States

**Keywords:** California conservation genomics project, California endangered species, CCGP, grasses, Poaceae, vernal pools

## Abstract

Colusa grass, *Neostapfia colusana*, is a listed California endangered plant endemic to the vernal pools of California. Vernal pool habitat is highly degraded and threatened by further anthropological development, with only 10% of its historical range remaining. With only 42 confirmed extant populations, it is a major conservation concern to understand patterns of genomic diversity. Here we report the first complete genome assembly of Colusa grass. The assembly includes two haplotypes: haplotype one spans 2.13 Gb with contig N50 of 10.62 Mb, scaffold N50 of 112.31 Mb, and BUSCO completeness of 98.1%. Haplotype two spans 2.04 Gb with contig N50 of 10.05 Mb and scaffold N50 of 138.31 Mb, with a BUSCO completeness of 97.6%. This genome assembly will allow for in-depth analysis of genomic variation and gene flow in populations of this threatened grass and will be a major asset to studies supporting its conservation. This genome was assembled as part of the California Conservation Genomics Project, which contributes to a collection of resources and tools to support state-wide conservation efforts.

## Introduction

Vernal pools are a unique type of seasonal wetlands and in California they are found primarily in the Great Central Valley. Given conversion of most of the valley to agriculture, California vernal pool habitat is now heavily degraded, with less than 10% of historical habitat remaining (Keeler-Wolf et al., 1998; Witham et al., 2013). Vernal pools are home to a wide assortment of unique plant and animal species (Keeley and Zedler 1998) due their unique ecology (Wellborn et al., 1996). Vernal pool grasses comprise some of the ~ 100 species of plants restricted to California vernal pools (Keeley and Zedler 1998) and are uniquely adapted to the soil type and seasonality of flooding and drought that characterize this extremely specialized habitat. These grasses are already rare due to their habitat specificity and are further endangered by climate change and habitat loss, with 94% of California vernal pool habitat already extirpated because of urbanization and agricultural expansion.

**Fig. 1:**
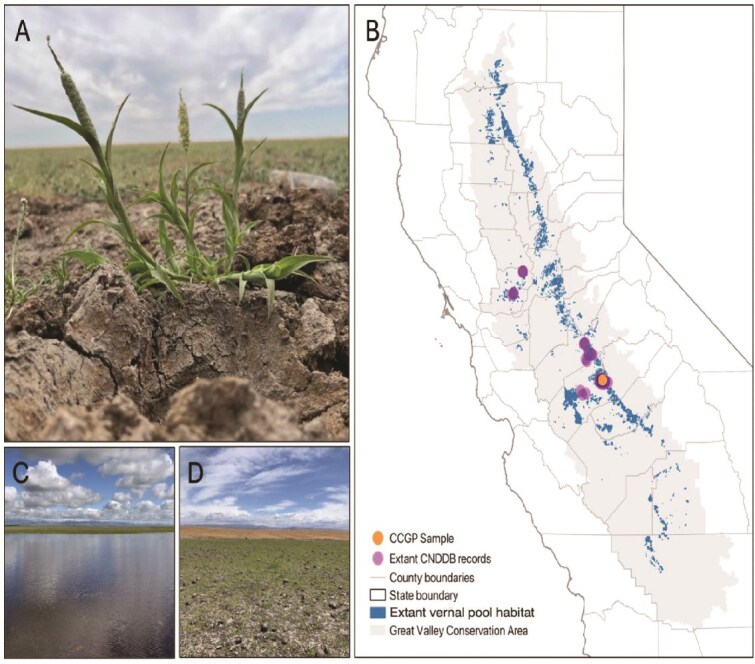
(A) *Neostapfia colusana* individual growing within a large population. (B) Map of vernal pool habitat in California, showing California natural diversity database (CNDDB) records of extant *N. colusana* populations and the location of the population used to source the reference genome sample. (C) Inundated vernal pool in Merced County. (D) the same vernal pool shown in panel C, after drying down, with a large population of *N. colusana*. *Photo credit: Dr. Daniel Toews.*

*Neostapfia colusana* (Poaceae; common name Colusa grass; Fig. 1) is endemic to California vernal pools (Keeley 1998) and is listed as endangered in the state of California (CNDDB 2023) and is federally listed as threatened (USFWS 2005). It is a member of the Cynodonteae tribe (subtribe Orcuttiinae) and is the only member of the genus *Neostapfia* (Reeder 1965). *Neostapfia colusana* is an annual, self-compatible, wind pollinated grass whose life history is linked with vernal pool ecology: germination occurs when pools are inundated, and juvenile leaves form underwater. As the pools dry the plant matures and forms terrestrial leaves (Keeley, 1998). It has a strong seed bank (Gordon et al., 2012), which allows for populations to remain dormant until more favorable conditions arise. Colusa grass does not establish upland of the vernal pools, suggesting it is a weak competitor (Keeley, 1998), however, this does mean that the loss of vernal pools results in the complete loss of habitat. There are only 42 known populations of *N. colusana*, down from 63 in 2006 (Gordon et al., 2012), with a majority of populations occurring on private land.

Despite its rarity and threatened status, genetic resources for *N. colusana* remain limited. Microsatellite markers revealed high genetic diversity within populations, and low gene flow among populations (Gordon et al., 2012). However, it remains unclear how much genetic variation is available in the seed bank, how this variation may vary from year to year, or if there is variation available to respond to rapid climate change. The availability of modern genomic resources, including a reference genome, will aid in *N. colusana* conservation, allowing genetic and genomic studies that further our understanding of adaptative potential and variation in this species, as well as ecologically similar plants.

Here, we present a high-quality genome assembly for *N. colusana* that we developed as a part of the California Conservation Genomics Project (CCGP, Shaffer et al. 2022), which is primarily concerned with landscape genomic variation patterns and trends across the state of California (Fiedler et al., 2022; Shaffer et al., 2022).

## Methods

To develop the high-quality genome assembly for *N. colusana* we sampled leaf tissue from live plants found during field surveys on the Flying M Ranch, an important vernal landscape that lies within the Southern Sierra Foothills Vernal Pool Region in East Merced County, CA (USFWS, 2005). The youngest leaves of the most robust plant were collected and flash-frozen in the field using liquid nitrogen. Leaf tissue was split into sterile 15-mL conical tubes and submerged in a liquid nitrogen bath until frozen. Tubes containing flash-frozen leaf material were immediately placed in dry ice and transported to the Sexton Lab at UC Merced where they were stored at −80°C. Frozen samples were shipped on dry ice to UC Davis (PacBio HiFi) and UC Santa Cruz (Dovetail Omni-C) for extraction and library preparation.

### DNA extraction

We extracted high molecular weight (HMW) genomic DNA from 81 mg of inflorescence tissue using the cetyltrimethylammonium bromide (CTAB) method as described in Inglis (2018), with the following modifications: (i) we used sodium metabisulfite (1% w/v) instead of 2-mercaptoethanol (1% v/v) in the sorbitol wash buffer and CTAB solution; (ii) we repeated the tissue homogenate wash steps until the supernatant turned clear; (iii) we performed the CTAB lysis step at 45°C and (iv) the chloroform extraction step twice using ice-cold chloroform. A majority of the shorter DNA fragments (<20 kilobases (kb)) from the resulting DNA were removed using the Short Read Eliminator-XL kit (Pacific Biosciences - PacBio, Menlo Park, CA) following the manufacturer’s instructions to enrich HMW DNA. The DNA purity was estimated by absorbance ratios (260/280 = 1.86 and 260/230 = 2.41) measured using the NanoDrop ND-1000 spectrophotometer (Thermo Fisher Scientific, Waltham, MA). The DNA yield (17.4 μg) was quantified using a Quantus Fluorometer (QuantiFluor ONE dsDNA Dye assay; Promega, Madison, WI), and the size distribution of the DNA was estimated using the Femto Pulse system (Genomic DNA 165 kb kit, Agilent, Santa Clara, CA), where 82% of the DNA fragments were found to be 20 kb or longer.

### Nucleic acid library preparation

#### Omni-C library

The Omni-C library was prepared using the Dovetail Omni-C Kit (Dovetail Genomics, Scotts Valley, CA) according to the manufacturer’s protocol with slight modifications. First, specimen tissue (Sample ID: NECOFM4.2) was thoroughly ground with a mortar and pestle while cooled with liquid nitrogen. Nuclear isolation was then performed using methods in Workman et al. (2018). Subsequently, chromatin was fixed in place in the nucleus and digested under various conditions of DNase I until a suitable fragment length distribution of DNA molecules was obtained. Chromatin ends were repaired and ligated to a biotinylated bridge adapter followed by proximity ligation of adapter-containing ends. After proximity ligation, crosslinks were reversed, and the DNA was purified from proteins. Purified DNA was treated to remove biotin that was not internal to ligated fragments. A next-generation sequencing (NGS) library was generated using an NEB Ultra II DNA Library Prep kit (New England Biolabs, Ipswich, MA) with an Illumina compatible y-adaptor. Biotin-containing fragments were then captured using streptavidin beads. The post capture product was split into two replicates prior to polymerase chain reaction (PCR) enrichment to preserve library complexity with each replicate receiving unique dual indices. The library was sequenced at Vincent J. Coates Genomics Sequencing Lab (Berkeley, CA) on an Illumina NovaSeq 6000 platform (Illumina, CA) to generate approximately 100 million 2 x 150 bp read pairs per gigabase (Gb) of genome size.

#### PacBio HiFi library preparation and sequencing

The HiFi SMRTbell library was constructed using the SMRTbell prep kit 3.0 (PacBio; Cat. #102–182-700) according to the manufacturer’s instructions. HMW gDNA was sheared to a target DNA size distribution between 15 and 18 kb using Diagenode’s Megaruptor 3 system (Diagenode, Belgium; cat. B06010003). The sheared gDNA was concentrated using 1X of SMRTbell cleanup beads provided in the SMRTbell prep kit 3.0 for the repair and a-tailing incubation at 37°C for 30 min and 65°C for 5 min, followed by ligation of overhang adapters at 20°C for 30 min, clean-up using 1X SMRTbell cleanup beads, and nuclease treatment at 37°C for 15 min. The SMRTbell library was size-selected using 3.1X of 35% v/v diluted AMPure PB beads (PacBio, Cat. #100–265-900) to progressively remove SMRTbell templates < 5 kb. The 15–18 kb average HiFi SMRTbell library was sequenced at UC Davis DNA Technologies Core (Davis, CA) using two 8 M SMRT cells (PacBio, Cat #101–389-001), Sequel IIe sequencing chemistry 2.0, and 30-h movies each on a PacBio Sequel II sequencer.

### Transcriptome library preparation and sequencing

Total RNA from 4 tissues (flowers, roots, stem, and leaves) was extracted using a Qiagen RNeasy Mini Kit (Qiagen, Netherlands), according to the manufacturer’s protocol. RNA libraries were then prepared using the KAPA mRNA HyperPrep Kit (Roche, Switzerland) according to the manufacturer’s protocol. Libraries were sequenced with 100 bp reads on an Illumina NovaSeq 6000 platform (Illumina, San Diego, CA), to generate approximately 50 M reads per library.

### Nuclear genome assembly

We assembled the genome of *N. colusana* following the CCGP assembly pipeline, which uses PacBio HiFi reads and Omni-C data to produce high quality and highly contiguous genome assemblies. The pipeline is outlined in Table 1 and lists the tools and non-default parameters used in the assembly process. First, we removed the remnant adapter sequences from the PacBio HiFi dataset using HiFiAdapterFilt (Sim et al., 2022) and generated the initial phased diploid assembly using HiFiasm (Cheng et al., 2021, 2022, 2024) in Hi-C mode, with the filtered PacBio HiFi reads and the Omni-C dataset. We aligned the Omni-C data separately to each assembly following the Arima Genomics Mapping Pipeline (https://github.com/ArimaGenomics/mapping_pipeline) and then scaffolded both assemblies with SALSA (Ghurye et al., 2017, 2019).

**Table 1:** Assembly pipeline and software used. Software citations are listed in the text.

**Assembly**	**Software and any non-default options**	**Version**
Filtering PacBio HiFi adapters	HiFiAdapterFilt	Commit 64d1c7b
K-mer counting	Meryl (k = 21)	1
Estimation of genome size and heterozygosity	GenomeScope	2
De novo *assembly (contiging)*	HiFiasm (Hi-C Mode, −primary, output p_ctg.hap1, p_ctg.hap2)	0.16.1-r375
**Scaffolding**		
Omni-C data alignment	Arima Genomics Mapping Pipeline	Commit 2e74ea4
Omni-C Scaffolding	SALSA (-DNASE, −i 20, −p yes)	2
Gap closing	YAGCloser (−mins 2 -f 20 -mcc 2 -prt 0.25 -eft 0.2 -pld 0.2)	Commit 0e34c3b
**Omni-C Contact map generation**		
Short-read alignment	BWA-MEM (-5SP)	0.7.17-r1198-dirty
SAM/BAM processing	samtools	1.16–8-g629fb5e (using htslib 1.16–3-g5036186)
SAM/BAM filtering	pairtools	0.3.0
Pairs indexing	pairix	0.3.7
Matrix generation	cooler	0.8.10
Matrix balancing	hicExplorer (hicCorrectmatrix correct --filterThreshold −2 4)	3.6
Contact map visualization	HiGlass	2.1.11
	PretextMap	0.1.4
	PretextView	0.1.5
	PretextSnapshot	0.0.3
Manual curation tools	Rapid curation pipeline (Wellcome Trust Sanger Institute, Genome Reference Informatics Team)	Commit 4ddca450
**Genome quality assessment**		
Basic assembly metrics	QUAST (−-est-ref-size)	5.0.2
Assembly completeness	BUSCO (−m geno, −l embryophyta)	5.0.0
	Merqury	2020 January 29
**Chloroplast genome assembly**		
de novo assembler of organelle genomes	Oatk	1.0 (Commit 6591c42)
Genome annotation	GeSeq (https://chlorobox.mpimp-golm.mpg.de/geseq.html)	Last accessed April 17th, 2025
**Contamination screening**		
Local alignment tool	BLAST+ (−db nt, −outfmt ‘6 qseqid staxids bitscore std’, −max_target_seqs 1, −max_hsps 1, −evalue 1e-25)	2.10
General contamination screening	BlobToolKit (PacBIo HiFi Coverage, NCBI Taxa ID = 160 571, BUSCODB = embryophyta)	2.3.3

Assemblies were manually curated by iteratively generating and analyzing their corresponding Omni-C contact maps. To generate the contact maps we aligned the Omni-C data to each assembly with BWA-MEM (Li 2013), identified ligation junctions, and generated Omni-C pairs using pairtools (Goloborodko et al., 2018). We generated a multi-resolution Omni-C matrix with cooler (Abdennur and Mirny, 2020) and balanced it with hicExplorer (Ramírez et al., 2018). We used HiGlass (Kerpedjiev et al., 2018) and the PretextSuite (https://github.com/wtsi-hpag/PretextView; https://github.com/wtsi-hpag/PretextMap; https://github.com/wtsi-hpag/PretextSnapshot) to visualize the contact maps where we identified misassemblies and misjoins, and finally modified the assemblies using the Rapid Curation pipeline from the Wellcome Trust Sanger Institute, Genome Reference Informatics Team (https://gitlab.com/wtsi-grit/rapid-curation). Some of the remaining gaps (joins generated during scaffolding and/or curation) were closed using the PacBio HiFi reads and YAGCloser (https://github.com/merlyescalona/yagcloser). Finally, we checked for contamination using the BlobToolKit Framework (Challis et al., 2020).

### Genome quality assessment

We generated k-mer counts from the PacBio HiFi reads using meryl (https://github.com/marbl/meryl). The k-mer counts were then used in GenomeScope2.0 (Ranallo-Benavidez et al., 2020) to estimate genome features including genome size, heterozygosity, and repeat content. For contiguity metrics, we ran QUAST (Gurevich et al., 2013). To evaluate genome quality and functional completeness we used BUSCO (Manni et al., 2021) with the Embryophyta ortholog database (embryophyta_odb10), which contains 1614 genes. Assessment of base level accuracy (QV) and k-mer completeness was performed using the previously generated meryl database and merqury (Rhie et al., 2020). We further estimated genome assembly accuracy via BUSCO gene set frameshift analysis using the pipeline described in Korlach et al. (2017). Measurements of the size of the phased blocks are based on the size of the contigs generated by HiFiasm in HiC mode. We follow the quality metric nomenclature established by Rhie et al. (2021), with the genome quality code x.y.P.Q.C, where, x = log10[contig NG50]; y = log10[scaffold NG50]; P = log10 [phased block NG50]; Q = Phred base accuracy QV (quality value); C = % genome represented by the first ‘n’ scaffolds, following a karyotype of 2n = 40, known for the number of chromosomes for this species (Genome on a Tree—GoaT; tax_tree(*N. colusana*); Challis et al., 2023). Quality metrics for the notation were calculated on the assembly for haplotype 1.

### Genome annotation

We annotated the reference assembly for this species using the NCBI Eukaryotic Genome Annotation Pipeline v0.3.2-alpha (hereafter, ‘EGAPx’) which is published in the NCBI RefSeq database (O’Leary et al. 2016) and accessible through the NCBI Github page (‘ncbi/egapx’). Annotation features were identified by aligning transcripts and proteins from related taxa in the RefSeq database using BLAST (Camacho et al., 2009). Novel, species-specific RNA-Seq reads generated from five tissue types were also aligned to the assembly using the alignment software STAR (Dobin et al. 2013). Additional features are predicted using HMM-based gene models using the NCBI Gnomon software. We evaluated the quality and completeness of our annotation by comparing the longest protein for each annotated coding gene to Eukaryotes and Poales (odb12) using BUSCO v5.8.2 (Manni et al. 2021). We report the number of annotation features and the BUSCO results in Table 2.

**Table 2:** Sequencing and assembly statistics, and accession numbers.

Bio Projects & Vouchers	CCGP NCBI BioProject			PRJNA720569			
	Genera NCBI BioProject			PRJNA765641			
	Species NCBI BioProject			PRJNA808353			
	NCBI Genome BioSample			SAMN35669218			
	Specimen identification (Genome)			DT0270			
	NCBI RNA BioSamples			SAMN41406410, SAMN41406412, SAMN41406413, SAMN41406411			
	NCBI Genome accessions			**Haplotype 1**		**Haplotype 2**	
	Assembly accession			JASWHV000000000		JASWHW000000000	
	Genome sequences			GCA_030760725.1		GCA_030760685.1	
Genome Sequence	PacBio HiFi reads		Run	1 PACBIO_SMRT (Sequel II) run: 5 M spots, 63.2G bases, 36.8Gb			
			Accession	SRX21418130			
	Omni-C Illumina reads		Run	2 ILLUMINA (Illumina NovaSeq 6000) runs: 64.4 M spots, 19.2G bases, 6.2Gb			
			Accession	SRX21418131, SRX21418132			
Genome Assembly Quality Metrics	Assembly identifier (Quality code^a^)				lpNeoColu1(7.8.P7.Q65.C95)		
	HiFi Read coverage^b^			28.55X			
				**Haplotype 1**		**Haplotype 2**	
	Number of contigs			1066		903	
	Contig N50 (bp)			10 620 575		10 054 094	
	Contig NG50^b^			10 111 628		8 846 118	
	Longest Contigs			47 898 884		35 341 938	
	Number of scaffolds			746		496	
	Scaffold N50			112 312 721		99 097 531	
	Scaffold NG50^b^			112 312 721		98 265 224	
	Largest scaffold			143 973 221		138 311 177	
	Size of final assembly			2 135 834 059		2 041 886 818	
	Phased block NG50^b^			10 880 489		9 559 241	
	Gaps per Gbp (# Gaps)			150(320)		199(407)	
	Indel QV (Frame shift)			47.89580712		46.43452676	
	Base pair QV			65.954		65.9425	
						Full assembly = 65.9484	
	k-mer completeness			87.666		86.5013	
						Full assembly = 97.0475	
	BUSCO completeness^c^ (embryophyta) *n* = 1614		**C**	**S**	**D**	**F**	**M**
		H1^d^	98.10%	85.60%	12.50%	0.60%	1.30%
		H2^d^	97.60%	86.00%	11.60%	0.60%	1.80%
	Organelles		1 complete chloroplast sequence			PV536199	
Genome annotation Quality metrics			Count of features				
	Genes		30 836				
	Transcripts		3706				
	mRNA		33 367				
	lncRNA		2410				
	CDSs		33 367				
	BUSCO completeness^d^		**C**	**S**	**D**	**F**	**M**
	Eukaryota (odb_12; *n* = 129)		100%	69.0%	31.0%	0.0%	0.0%
	Poales (odb_12; *n* = 6282)		96.3%	69.4%	26.9%	0.9%	2.8%

a Assembly quality code x.y.P.Q.C derived notation, from (Rhie et al. 2021). x = log10[contig NG50]; y = log10[scaffold NG50]; P = log10 [phased block NG50]; Q = Phred base accuracy QV (Quality value); C = % genome represented by the first ‘n’ scaffolds, following a karyotype of 2n = 40, known for the number of chromosomes for this species (Genome on a Tree—GoaT; tax_tree(*Neostapfia colusana*); Challis et al. 2023). Quality metrics for the notation were calculated on the assembly for haplotype 1.

b Read coverage and NGx statistics have been calculated based on the estimated genome size of 2.21 Gb

c (H1) Haplotype 1 and (H2)Haplotype 2 assembly values.

d BUSCO Scores. Complete BUSCOs (C). Complete and single-copy BUSCOs (S). Complete and duplicated BUSCOs (D). Fragmented BUSCOs (F). Missing BUSCOs (M).

### Chloroplast genome assembly

The chloroplast sequence for *N.colusana* was generated with Oatk (https://github.com/c-zhou/oatk, Zhou et al., 2024). We used the chloroplast genome assembly from *Arabidopsis thaliana* (NCBI:NC_000932.1; Sato et al., 1999) as a guide for manual curation, in which we aligned the generated sequence against the guide, using lastz (Harris, 2007), extracted contigs and fixed orientation when needed using samtools (Danecek et al., 2021) and seqtk (https://github.com/lh3/seqtk). Visual validation of the alignment was done using LAJ (Wilson et al., 2001). The resulting assembly was annotated using the online version of GeSeq (Tillich et al., 2017) and visualized using the online version of OGDRAW (Greiner et al., 2019).

## Results

**Fig. 2:**
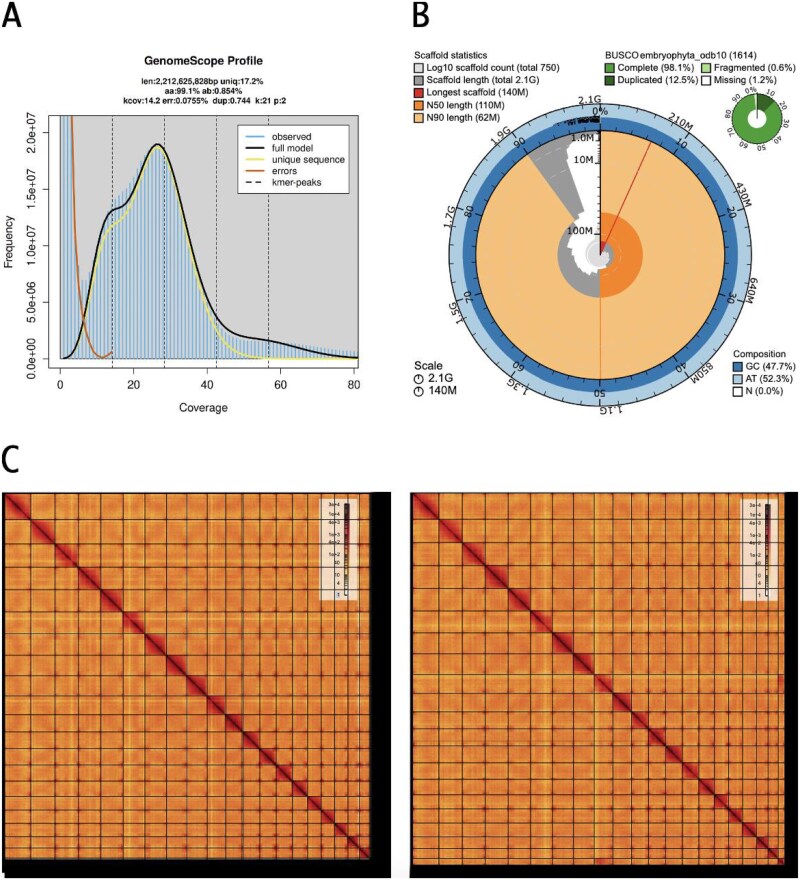
(A) *K-mer frequency distribution as a function of coverage*. Horizontal bars represent the observed k-mer frequencies, while the curved lines show the GenomeScope model fit and the contributions of unique sequences and sequencing errors. Vertical dashed lines mark estimated k-mer coverage peaks. (B) *Summary assembly metrics for haplotype 1 assembly:* The circular plot presents scaffold statistics, with scaffold lengths and scaffold counts (log10 scale). Outer rings show GC content (47.7%) and AT content (52.3%), with no ambiguous bases (N). The BUSCO assessment (inset) indicates high assembly completeness. (C) *Omni-C interaction heatmap*s are shown for haplotype 1 (left) and haplotype 2 (right) assemblies at 10 kb resolution, visualized at a current resolution of 5.12 mb. Each heatmap reflects chromatin interaction frequency, with darker shades representing higher contact intensity. Strong diagonal patterns and distinct chromosomal interaction blocks indicate well-assembled, chromosome-scale scaffolds. The maps support the structural integrity of both assemblies and highlight the continuity and organization of genomic regions in the *lpNeoColu1* genome.

The Omni-C and PacBio HiFi sequencing libraries generated 63.37 million read pairs and 4.96 million reads respectively. The latter yielded ~ 28-fold coverage, with anN50 read length 14 330 bp; minimum read length 154 bp; mean read length 12 772 bp; maximum read length of 64 026 bp (Fig. S1). Based on the PacBio HiFi data, Genomescope 2.0 estimated a genome size of 2.21 Gb, a 0.0755% sequencing error rate and 0.854% nucleotide heterozygosity rate. The k-mer spectrum based on PacBio HiFi reads show a bimodal distribution with two major peaks at ~ 14 and ~ 28-fold coverage (Fig. 2A). Sequencing of mRNA libraries for SAMN41406410, SAMN41406412, SAMN41406413, and SAMN41406411 yielded 34 M, 41 M, 54 M, and 60 M paired-end reads, respectively.

The final assembly (lpNeoColu1) consists of two phased haplotypes that vary slightly in size compared with the estimated value from GenomeScope2.0 (Fig. 2A), as has been observed in other taxa (see Pflug et al., 2020). Haplotype one consists of 746 scaffolds spanning 2.13 Gb with contig N50 of 10.62 Mb, scaffold N50 of 112.31 Mb, largest contig of 47.89 Mb and largest scaffold of 143.97 Mb. Haplotype two consists of 496 scaffolds, spanning 2.04 Gb with contig N50 of 10.05 Mb, scaffold N50 of 138.31 Mb, largest contig 35.34 Mb and largest scaffold of 138.31 Mb.

During manual curation, we generated a total of 54 breaks and 340 joins; where 23 breaks were made on haplotype one and 31 were made on haplotype two, whereas we made 149 joins on haplotype one, and 191 joins on haplotype two. We were able to close a total of 82 gaps, 32 on haplotype one and 50 on haplotype two. No further modifications were made to our assemblies.

Haplotype one has a BUSCO completeness score of 98.1% using the Embryophyta gene set, a per-base quality (QV) of 65.95, a kmer completeness of 87.66 and a frameshift indel QV of 47.89. The haplotype two has a BUSCO completeness score of 97.6% using the same gene set, a per base quality (QV) of 65.94, a kmer completeness of 86.5 and a frameshift indel QV of 46.43. Assembly statistics are reported in Table 2, and graphical representation for the haplotype 1 assembly in Fig. 2B.

The Omni-C contact maps show highly contiguous assemblies, with chromosome-length scaffolds, suggesting that the *N. colusana* genome is organized in 20 chromosomes based on the number of major bins along the diagonal axis of the plot, and the chromosome-scaffolds have been named based on their ordered length (Fig. 2C, 2D, Supplementary Table 1). N. colusana appears to be diploid (2n = 40), as reported first in Reeder (1982), which is unique in this family (Hilu 2004), but common in this tribe (Reeder 1982). Assembly statistics are reported in Table 2 and represented graphically in Fig. 2B. We have deposited the genome assembly on NCBI GenBank (See Table 2 and Data Availability for details).

Our final genome annotation for *N. colusana* included 30 836 genes, with a Poales BUSCO completeness of 96.3%. A list of annotation statistics and BUSCO score breakdowns for Eukaryota and Poales gene sets is reported in Table 2.

### Chloroplast genome assembly

The final chloroplast genome assembly spans 135 891 bp, has a nucleotide composition of A = 31.03%, C = 19.03%, G = 19.17%, T = 30.77%. The large single copy is 81 437 bp long, the small single copy is 12 551 bp long, and the inverted repeat is 20 950 bp long (Fig. S2). The chloroplast sequence has been deposited on NCBI (see Data Availability for details).

## Discussion

To our knowledge, this is the first genome published for a plant species restricted to vernal pool habitats, and as such, is also the first published genome for this subtribe of grasses (tribe Cynodonteae, subtribe Orcuttiinae). *Neostapfia colusana* and other vernal pool plant populations are in decline due to habitat degradation and changing climates. Within subtribe Orcuttiinae, several species have evolved and specialized in the unique hydrology of vernal pool ecosystems (Reeder, 1965), yet due to modern land changes, all species within this group are now listed by the State of California or the Federal Government as endangered or threatened. Habitat restoration and population management will be crucial for the persistence of these species, and these vernal pool grasses serve as key indicators for success in restoration efforts of endangered wetlands in California.

California vernal pool ecosystems are endangered yet harbor a great number of unique and endemic species that California and the US Fish and Wildlife Services are charged to protect and manage. Restoration efforts are ongoing and can benefit from predicting and mapping genetic diversity. This reference genome will be highly valuable for population genomics studies that can target quality and function of genomic diversity across the species’ range. The use of a high-quality reference genome in these studies will aid in the development of improved genetic tools and increased understanding of the distribution of genetic diversity within California vernal pool ecosystems.

The availability of this reference genome, and the landscape genomic patterns to come from the next CCGP efforts, will open this species up to more avenues of research, which will aid in its management and persistence. The last genetic assessment of this species, based on microsatellite markers, found high rates of genetic variation with little connectivity between northern and southern populations (Gordon et al., 2012). Updated studies using the new reference genome and sampling across the range will allow for a more complete understanding of past and current trends in genomic variation and gene flow, which can inform management and restoration decisions. Furthermore, the destruction of vernal pool habitat in recent years is often required to be mitigated with the creation of new habitat; that is to say, human-made pools that can become habitat for vernal pool species. Using these genomic tools, researchers will be able to better understand what genotypes can be used for mitigation, and if these synthetic pools differ over time from natural pools in terms of the genetic diversity they can support. Further, these updated genomic tools can be used in research into the adaptability of current populations, and how these populations may or may not be able to respond to climate change.

Understanding and managing vernal pool grasses benefits myriad other wetland species, including other plants, animals, fungi, and microbes in these ecosystems (Ruiz-Ramos et al., 2023). Moreover, past populations can inform current populations to further understand vernal pool habitat. Historical samples from now extirpated *N. colusana* populations are available in herbaria, providing the opportunity to understand past and present gene flow and genomic variation in this species, and to understand what massive population loss does to a species. These samples can also help inform current restoration strategies— estimating past rates of gene flow and habitat connectivity can inform current restoration activities (Proft et al., 2018) and provide a baseline for these activities to work toward. This work will enhance and complement other projects and species in the CCGP (e.g., *Orcuttia* and *Tuctoria* species) and management programs in California by establishing genomic baselines that can be used by management to set priorities and goals across endangered ecosystems such as those found in the California Great Central Valley.

## Supplementary Material

Supplementary_Material_esaf075

## Data Availability

Data generated for this study are available under NCBI BioProject PRJNA808353. Raw sequencing data for sample DT0270 (NCBI BioSample SAMN35669218) are deposited in the NCBI Short Read Archive (SRA) under SRR25693254 for PacBio HiFi sequencing data, and SRR25693252–53 for the Omni-C Illumina sequencing data. GenBank accessions for both Haplotype 1 and Haplotype 2assemblies are GCA_030760725.1 and GCA_030760685.1; and for genome sequences JASWHV000000000 and JASWHW000000000. The GenBank accession for the chloroplast genome is PV536199. Assembly scripts and other data for the analyses presented can be found at the following GitHub repository: www.github.com/ccgproject/ccgp_assembly. Data generated for the annotation in this study are available under NCBI BioProject PRJNA1013274. Raw RNA sequencing data (NCBI BioSample(s) SAMN41406410, SAMN41406412, SAMN41406413, SAMN41406411) are deposited in the NCBI SRA [SRR29299416, SRR29299414, SRR29299413, SRR29299415], respectively). Genome annotation output is stored on Dryad, dataset DOI: 10.5061/dryad.b8gtht7rn.

## References

[ref1] Abdennur N, Mirny LA. Cooler: scalable storage for hi-C data and other genomically labeled arrays. Bioinformatics. 2020;36:311–316. 10.1093/bioinformatics/btz540 31290943 PMC8205516

[ref2] California Natural Diversity Database (CNDDB) . State and federally listed endangered, threatened, and rare plants of California. Sacramento, CA: California Department of Fish and Wildlife; 2023. https://nrm.dfg.ca.gov/FileHandler.ashx?DocumentID=109390&inline

[ref2a] Camacho C, Coulouris G., Avagyan V, Ma N, Papadopoulos J, Bealer K, Madden TL. BLAST+: architecture and applications. BMC Bioinformatics. 2009;10:421. 10.1186/1471-2105-10-421 PMC2803857 20003500

[ref3] Challis R, Kumar S, Sotero-Caio C, Brown M, Blaxter M. Genomes on a tree (GoaT): a versatile, scalable search engine for genomic and sequencing project metadata across the eukaryotic tree of life. Wellcome Open Res. 2023;8:24. 10.12688/wellcomeopenres.18658.1 36864925 PMC9971660

[ref4] Challis R, Richards E, Rajan J, Cochrane G, Blaxter M. BlobToolKit – interactive quality assessment of genome assemblies. G3 GenesGenomesGenetics. 2020;10:1361–1374. 10.1534/g3.119.400908 PMC7144090 32071071

[ref5] Cheng H, Asri M, Lucas J, Koren S, Li H. Scalable telomere-to-telomere assembly for diploid and polyploid genomes with double graph. Nat Methods. 2024;21:967–970. 10.1038/s41592-024-02269-8 38730258 PMC11214949

[ref6] Cheng H, Concepcion GT, Feng X, Zhang H, Li H. Haplotype-resolved de novo assembly using phased assembly graphs with hifiasm. Nat Methods. 2021;18:170–175. 10.1038/s41592-020-01056-5 33526886 PMC7961889

[ref7] Cheng H, Jarvis ED, Fedrigo O, Koepfli K-P, Urban L, Gemmell NJ, Li H. Haplotype-resolved assembly of diploid genomes without parental data. Nat Biotechnol. 2022;40:1332–1335. 10.1038/s41587-022-01261-x 35332338 PMC9464699

[ref8] Danecek P, Bonfield JK, Liddle J, Marshall J, Ohan V, Pollard MO, Whitwham A, Keane T, McCarthy SA, Davies RM, et al. Twelve years of SAMtools and BCFtools. GigaScience. 2021;10:giab008. 10.1093/gigascience/giab008 33590861 PMC7931819

[ref9] Dobin A, Davis CA, Schlesinger F, Drenkow J, Zaleski C, Jha S, Batut P, Chaisson M, Gingeras TR. STAR: ultrafast universal RNA-seq aligner. Bioinformatics. 2013;29:15–21. 10.1093/bioinformatics/bts635 23104886 PMC3530905

[ref10] Fiedler PL, Erickson B, Esgro M, Gold M, Hull JM, Norris JM, Shapiro B, Westphal M, Toffelmier E, Shaffer HB. Seizing the moment: the opportunity and relevance of the California conservation genomics project to state and federal conservation policy. J Hered. 2022;113:589–596. 10.1093/jhered/esac046 36136001 PMC9709969

[ref11] Ghurye J, Pop M, Koren S, Bickhart D, Chin C-S. Scaffolding of long read assemblies using long range contact information. BMC Genomics. 2017;18:527. 10.1186/s12864-017-3879-z 28701198 PMC5508778

[ref12] Ghurye J, Rhie A, Walenz BP, Schmitt A, Selvaraj S, Pop M, Phillippy AM, Koren S. Integrating hi-C links with assembly graphs for chromosome-scale assembly. PLoS Comput Biol. 2019;15:e1007273. 10.1371/journal.pcbi.1007273 31433799 PMC6719893

[ref13] Goloborodko A, Abdennur N, Venev S, Brandao HB, Fudenberg G. mirnylab/pairtools: v0. 2.0. 2018. 10.5281/zenodo.1490831

[ref14] Gordon SP, Sloop CM, Davis HG, Cushman JH. Population genetic diversity and structure of two rare vernal pool grasses in Central California. Conserv Genet. 2012;13:117–130. 10.1007/s10592-011-0269-y

[ref15] Greiner S, Lehwark P, Bock R. OrganellarGenomeDRAW (OGDRAW) version 1.3.1: expanded toolkit for the graphical visualization of organellar genomes. Nucleic Acids Res. 2019;47:W59–W64. 10.1093/nar/gkz238 30949694 PMC6602502

[ref16] Gurevich A, Saveliev V, Vyahhi N, Tesler G. QUAST: quality assessment tool for genome assemblies. Bioinformatics. 2013;29:1072–1075. 10.1093/bioinformatics/btt086 23422339 PMC3624806

[ref17] Harris RS . Improved pairwise alignment of genomic DNA (Ph.D.). In: Pennsylvania: The Pennsylvania State University, United States. 2007.

[ref18] Hilu KW . Phylogenetics and chromosomal evolution in the Poaceae (grasses). Aust J Bot. 2004;52(1):13–22. 10.1071/BT03103

[ref19] Inglis PW, Pappas M d CR, Resende LV, Grattapaglia D. Fast and inexpensive protocols for consistent extraction of high quality DNA and RNA from challenging plant and fungal samples for high-throughput SNP genotyping and sequencing applications. PLoS One. 2018;13:e0206085. 10.1371/journal.pone.0206085 30335843 PMC6193717

[ref20] Keeler-Wolf T, Elam DR, Lewis K, Flint SA. California vernal pool assessment preliminary report. Fish Game Sacram: State Calif. Dep; 1998.

[ref21] Keeley JE . C4 photosynthetic modifications in the evolutionary transition from land to water in aquatic grasses. Oecologia. 1998;116:85–97. 10.1007/s004420050566 28308544

[ref22] Kerpedjiev P, Abdennur N, Lekschas F, McCallum C, Dinkla K, Strobelt H, Luber JM, Ouellette SB, Azhir A, Kumar N, et al. HiGlass: web-based visual exploration and analysis of genome interaction maps. Genome Biol. 2018;19:125. 10.1186/s13059-018-1486-1 30143029 PMC6109259

[ref23] Korlach J, Gedman G, Kingan SB, Chin C-S, Howard JT, Audet J-N, Cantin L, Jarvis ED. De novo PacBio long-read and phased avian genome assemblies correct and add to reference genes generated with intermediate and short reads. GigaScience. 2017;6:1–16. 10.1093/gigascience/gix085 PMC5632298 29020750

[ref24] Li H . Aligning sequence reads, clone sequences and assembly contigs with BWA-MEM. arXiv preprint arXiv:1303.3997. http://arxiv.org/abs/1303.3997

[ref25] Manni M, Berkeley MR, Seppey M, Simão FA, Zdobnov EM. BUSCO update: novel and streamlined workflows along with broader and deeper phylogenetic coverage for scoring of eukaryotic, prokaryotic, and viral genomes. Mol Biol Evol. 2021;38:4647–4654. 10.1093/molbev/msab199 34320186 PMC8476166

[ref26] O’Leary NA, Wright MW, Brister JR, Ciufo S, Haddad D, McVeigh R, et al. Reference sequence (RefSeq) database at NCBI: current status, taxonomic expansion, and functional annotation. Nucleic Acids Res. 2016;44:D733–D745. 10.1093/nar/gkv1189 26553804 PMC4702849

[ref27] Pflug JM, Holmes VR, Burrus C, Johnston JS, Maddison DR. Measuring genome sizes using read-depth, k-mers, and flow cytometry: methodological comparisons in beetles (coleoptera). G3 GenesGenomesGenetics. 2020;10:3047–3060. 10.1534/g3.120.401028 PMC7466995 32601059

[ref28] Proft KM, Jones ME, Johnson CN, Burridge CP. Making the connection: expanding the role of restoration genetics in restoring and evaluating connectivity. Restor Ecol. 2018;26:411–418. 10.1111/rec.12692

[ref28a] Ramírez F, Bhardwaj V, Arrigoni L, Lam KC, Grüning BA, Villaveces J, Habermann B, Akhtar A, Manke, T. High-resolution TADs reveal DNA sequences underlying genome organization in flies. Nat Commun. 2018;9. 10.1038/s41467-017-02525-w PMC5768762 29335486

[ref29] Ranallo-Benavidez TR, Jaron KS, Schatz MC. GenomeScope 2.0 and Smudgeplot for reference-free profiling of polyploid genomes. Nat Commun. 2020;11:1432. 10.1038/s41467-020-14998-3 32188846 PMC7080791

[ref30] Reeder JR . Systematics of the tribe Orcuttieae (gramineae) and the description of a new segregate genus, Tuctoria. Am J Bot. 1982;69:1082–1095. 10.1002/j.1537-2197.1982.tb13353.x

[ref31] Reeder JR . The tribe Orcuttieae and the subtribes of the Pappophoreae (gramineae). Madroño. 1965;18:18–28

[ref32] Rhie A, McCarthy SA, Fedrigo O, Damas J, Formenti G, Koren S, Uliano-Silva M, Chow W, Fungtammasan A, Kim J, et al. Towards complete and error-free genome assemblies of all vertebrate species. Nature. 2021;592:737–746. 10.1038/s41586-021-03451-0 33911273 PMC8081667

[ref33] Rhie A, Walenz BP, Koren S, Phillippy AM. Merqury: reference-free quality, completeness, and phasing assessment for genome assemblies. Genome Biol. 2020;21:245. 10.1186/s13059-020-02134-9 32928274 PMC7488777

[ref34] Ruiz-Ramos DV, Meyer RS, Toews D, Stephens M, Kolster MK, Sexton JP. Environmental DNA (eDNA) detects temporal and habitat effects on community composition and endangered species in ephemeral ecosystems: a case study in vernal pools. Environ DNA. 2023;5:85–101. 10.1002/edn3.360

[ref35] Sato S, Nakamura Y, Kaneko T, Asamizu E, Tabata S. Complete structure of the chloroplast genome of Arabidopsis thaliana. DNA Res. 1999;6:283–290. 10.1093/dnares/6.5.283 10574454

[ref36] Shaffer HB, Toffelmier E, Corbett-Detig RB, Escalona M, Erickson B, Fiedler P, Gold M, Harrigan RJ, Hodges S, Luckau TK, et al. Landscape genomics to enable conservation actions: the California conservation genomics project. J Hered. 2022;113:577–588. 10.1093/jhered/esac020 35395669

[ref37] Sim SB, Corpuz RL, Simmonds TJ, Geib SM. HiFiAdapterFilt, a memory efficient read processing pipeline, prevents occurrence of adapter sequence in PacBio HiFi reads and their negative impacts on genome assembly. BMC Genomics. 2022;23:157. 10.1186/s12864-022-08375-1 35193521 PMC8864876

[ref38] Tillich M, Lehwark P, Pellizzer T, Ulbricht-Jones ES, Fischer A, Bock R, Greiner S. GeSeq – versatile and accurate annotation of organelle genomes. Nucleic Acids Res. 2017;45:W6–W11. 10.1093/nar/gkx391 28486635 PMC5570176

[ref39] U.S. Fish and Wildlife Service . Recovery plan for vernal pool ecosystems of California and southern Oregon. Federal Register. Vol. xxvi. Portland, Oregon; 2005:+ 606.

[ref40] Wellborn GA, Skelly DK, Werner EE. MECHANISMS creating community structure across a freshwater habitat gradient. Annu Rev Ecol Evol Syst. 1996;27:337–363. 10.1146/annurev.ecolsys.27.1.337

[ref41] Wilson MD, Riemer C, Martindale DW, Schnupf P, Boright AP, Cheung TL, Hardy DM, Schwartz S, Scherer SW, Tsui L-C, et al. Comparative analysis of the gene-dense ACHE/TFR2 region on human chromosome 7q22 with the orthologous region on mouse chromosome 5. Nucleic Acids Res. 2001;29:1352–1365. 10.1093/nar/29.6.1352 11239002 PMC29746

[ref42] Witham C, Holland R, Vollmar J. Great Valley vernal pool map, plus Merced, placer and Sacramento County losses 2005–2010. Sacramento, CA; 2013: 2005: Report prepared for the U.S. Fish and Wildlife Service’s and Bureau of Reclamation’s CVPIA Habitat Restoration Program under Grant Agreement No. 80270-A-G509 with the USFWS.

[ref43] Workman R, Timp W, Fedak R, Kilburn D, Hao S, Liu K. High molecular weight DNA extraction from recalcitrant plant species for third generation sequencing. Adv Mater. 2018. 10.1038/protex.2018.059

[ref44] Zhou C, Brown M, Blaxter M, The Darwin Tree of Life Project Consortium, McCarthy SA, Durbin R. Oatk: a de novo assembly tool for complex plant organelle genomes. bioRxiv. 2024. 10.1101/2024.10.23.619857 PMC12329965 40775726

