# Supplementary material for: A reference genome for Colusa grass, Neostapfia colusana, a threatened and endangered California vernal pool plant
Source: J Hered. 2025 Oct 7;117(3):459–68. doi: 10.1093/jhered/esaf075 (PMC13147174; doi:10.1093/jhered/esaf075)
Supplement: Supplementary_Material_esaf075 [file supplementary_material_esaf075.docx]

# Supplementary Material

**Table S1.** Summary of haplotype assemblies for the *Neostapfia colusana* genome. Accession numbers, scaffold names, scaffold lengths, and chromosome assignments are shown for both haplotype 1 and haplotype 2 across all 20 chromosomes.

| **Name** | **Haplotype 1** | | | **Haplotype 2** | | |
| --- | --- | --- | --- | --- | --- | --- |
|  | **Accession Number** | **Scaffold name** | **Length** | **Accession Number** | **Scaffold name** | **Length** |
| Chr1 | JASWHV010000001.1 | SCAF_1 | 143,973,221 | JASWHW010000001.1 | SCAF_1 | 138,311,177 |
| Chr2 | JASWHV010000002.1 | SCAF_2 | 134,177,870 | JASWHW010000002.1 | SCAF_2 | 123,738,870 |
| Chr3 | JASWHV010000003.1 | SCAF_3 | 129,084,687 | JASWHW010000003.1 | SCAF_3 | 121,429,446 |
| Chr4 | JASWHV010000004.1 | SCAF_4 | 124,651,085 | JASWHW010000004.1 | SCAF_4 | 121,261,231 |
| Chr5 | JASWHV010000005.1 | SCAF_5 | 123,239,891 | JASWHW010000005.1 | SCAF_5 | 118,422,318 |
| Chr6 | JASWHV010000006.1 | SCAF_6 | 120,367,127 | JASWHW010000006.1 | SCAF_6 | 115,546,930 |
| Chr7 | JASWHV010000007.1 | SCAF_7 | 119,364,135 | JASWHW010000007.1 | SCAF_7 | 111,191,100 |
| Chr8 | JASWHV010000008.1 | SCAF_8 | 112,970,913 | JASWHW010000008.1 | SCAF_8 | 109,991,397 |
| Chr9 | JASWHV010000009.1 | SCAF_9 | 112,312,721 | JASWHW010000009.1 | SCAF_9 | 99,097,531 |
| Chr10 | JASWHV010000010.1 | SCAF_10 | 103,403,390 | JASWHW010000010.1 | SCAF_10 | 98,265,224 |
| Chr11 | JASWHV010000011.1 | SCAF_11 | 95,107,376 | JASWHW010000011.1 | SCAF_11 | 89,829,177 |
| Chr12 | JASWHV010000012.1 | SCAF_12 | 92,870,918 | JASWHW010000012.1 | SCAF_12 | 89,803,804 |
| Chr13 | JASWHV010000013.1 | SCAF_13 | 90,531,838 | JASWHW010000013.1 | SCAF_13 | 89,577,471 |
| Chr14 | JASWHV010000014.1 | SCAF_14 | 89,159,537 | JASWHW010000014.1 | SCAF_14 | 86,838,761 |
| Chr15 | JASWHV010000015.1 | SCAF_15 | 84,794,784 | JASWHW010000015.1 | SCAF_15 | 81,988,681 |
| Chr16 | JASWHV010000016.1 | SCAF_16 | 75,275,394 | JASWHW010000016.1 | SCAF_16 | 75,428,743 |
| Chr17 | JASWHV010000017.1 | SCAF_17 | 74,425,335 | JASWHW010000017.1 | SCAF_17 | 74,240,053 |
| Chr18 | JASWHV010000018.1 | SCAF_18 | 73,670,733 | JASWHW010000018.1 | SCAF_18 | 73,299,964 |
| Chr19 | JASWHV010000019.1 | SCAF_19 | 62,217,899 | JASWHW010000019.1 | SCAF_19 | 61,305,453 |
| Chr20 | JASWHV010000020.1 | SCAF_20 | 56,197,841 | JASWHW010000020.1 | SCAF_20 | 53,845,723 |

**
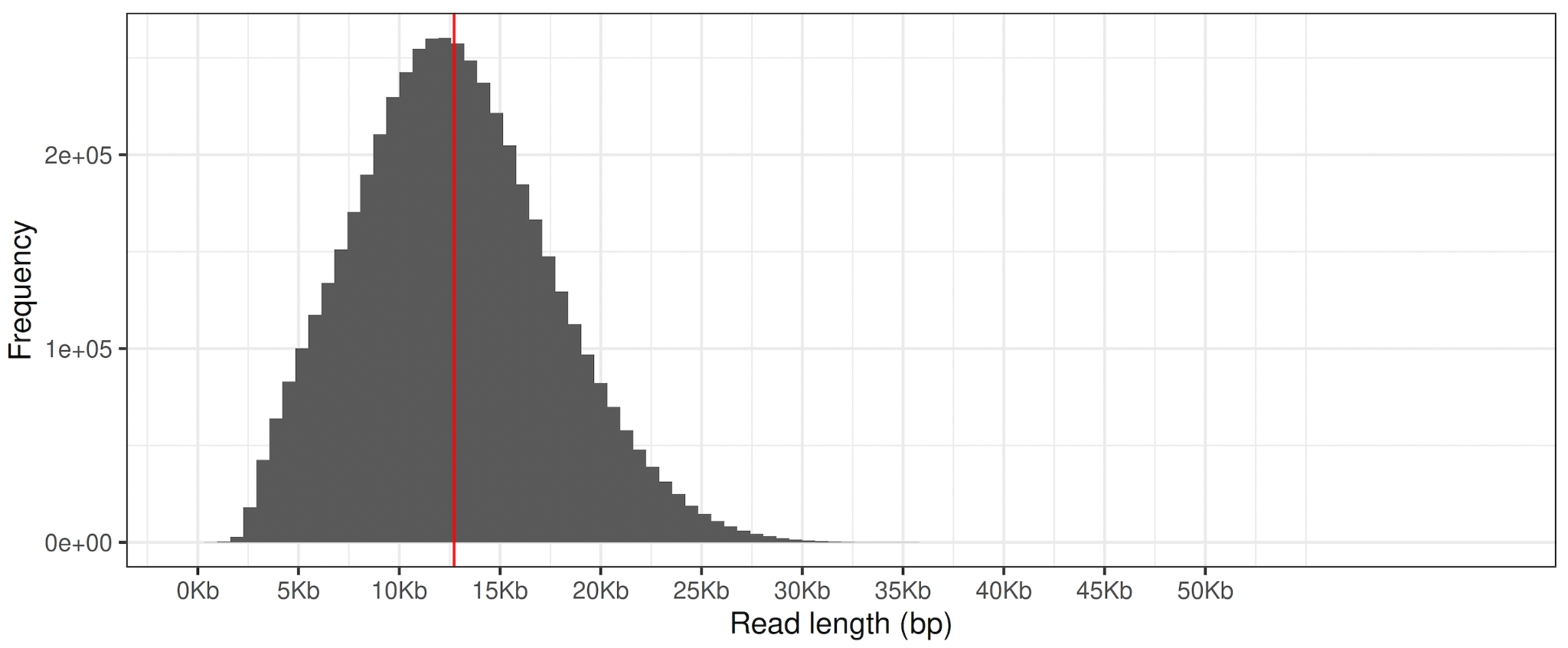
**

**Figure S1.** PacBio HiFi read length distribution.

**
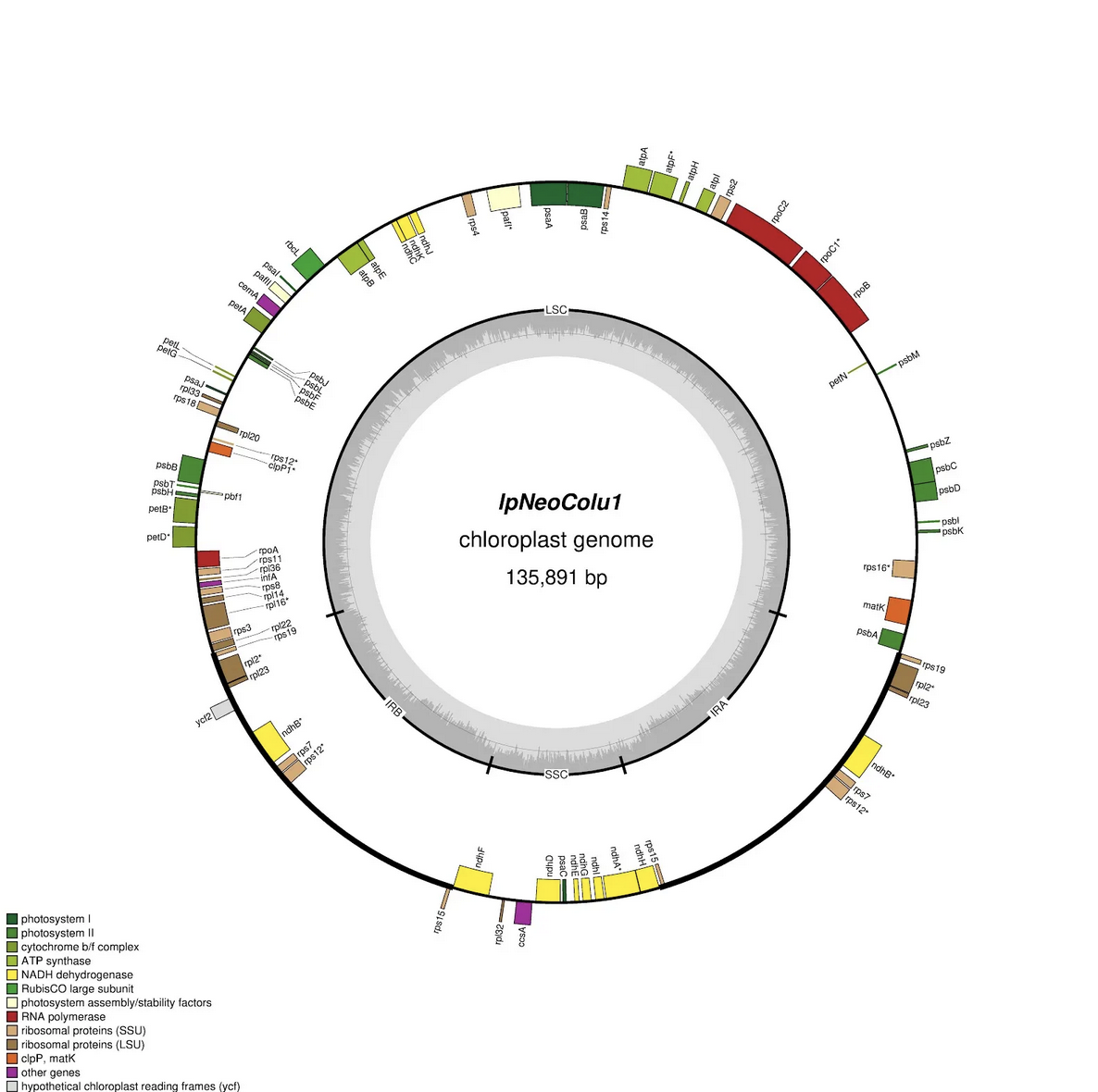
**

**Figure S2.** Final chloroplast genome assembly
